# Newly Engineered Magnetic Erythrocytes for Sustained and Targeted Delivery of Anti-Cancer Therapeutic Compounds

**DOI:** 10.1371/journal.pone.0017132

**Published:** 2011-02-23

**Authors:** Caterina Cinti, Monia Taranta, Ilaria Naldi, Settimio Grimaldi

**Affiliations:** 1 Institute of Clinical Physiology, Consiglio Nazionale delle Ricerche, Siena, Italy; 2 Institute of Translational Pharmacology, Consiglio Nazionale delle Ricerche, Rome, Italy; University of Louisville, United States of America

## Abstract

Cytotoxic chemotherapy of cancer is limited by serious, sometimes life-threatening, side effects that arise from toxicities to sensitive normal cells because the therapies are not selective for malignant cells. So how can they be selectively improved? Alternative pharmaceutical formulations of anti-cancer agents have been investigated in order to improve conventional chemotherapy treatment. These formulations are associated with problems like severe toxic side effects on healthy organs, drug resistance and limited access of the drug to the tumor sites suggested the need to focus on site-specific controlled drug delivery systems. In response to these concerns, we have developed a new drug delivery system based on magnetic erythrocytes engineered with a viral spike fusion protein. This new erythrocyte-based drug delivery system has the potential for magnetic-controlled site-specific localization and highly efficient fusion capability with the targeted cells. Here we show that the erythro-magneto-HA virosomes drug delivery system is able to attach and fuse with the target cells and to efficiently release therapeutic compounds inside the cells. The efficacy of the anti-cancer drug employed is increased and the dose required is 10 time less than that needed with conventional therapy.

## Introduction

The success of any medical treatment depends not only upon the pharmacokinetic/pharmacodynamic activity of the therapeutic agent, but to a large extent, on its bioavailability at the site of action in the human system [1]–[4]. In the past, alternative pharmaceutical formulations of anti-cancer agents have been investigated in order to improve conventional chemotherapy treatment. In conventional/current therapy oral tablet, capsule and injectable formulations are used for anti-cancer drugs delivery. These formulations are associated with problems like severe toxic side effects on healthy organs, difficulties in clinical administration, drug resistance and limited access of the drug to the tumor sites suggested the need to focus on site specific controlled drug delivery systems.

Drug delivery systems (DDSs) such as lipid- or polymer-based nanoparticles have been designed to improve the pharmacological and therapeutic properties of drugs administered parenterally. The majority of the DDS currently approved for parenteral administration falls into the category of liposomal or lipid-based formulations or therapeutic molecules linked to polyethylene glycol (PEG) [5]–[7]. Although drug solubility may not be a limiting factor for systems such as polymer-drug conjugates, in which the drug is chemically linked to the carrier, it can be an important consideration in liposomal DDS. Carrying capacity of liposomes is not efficient for very large therapeutic molecules such as proteins, particularly when small liposome diameters are desirable for reasons of biodistribution. Biodegradable nano/microparticles of poly(D,L-lactide-co-glycolide) (PLGA) and PLGA-based polymers are widely explored as carriers for controlled delivery of macromolecular therapeutics such as proteins, peptides, vaccines, genes, antigens and growth factors. Literature cites many advantages and drawbacks of PLGA and PLGA-based delivery systems for delivering macromolecular drugs [8], [9]. Drug encapsulation, particle size, additives added during formulation, molecular weight, ratio of lactide to glycolide moieties in PLGA and surface morphology could influence the release characteristics [10]. PLGA has a negative effect on protein stability during the preparation and storage, primarily due to the acid-catalyzed nature of its degradation [11]. In addition, processing conditions used in the manufacturing of PLGA drug delivery vehicles have detrimental effects on certain protein secondary structures [12].

In the past few years, cell-based delivery systems have also been developed. The use of cells as therapeutic carriers has developed as a distinct concept and delivery method [13]–[17]. Cell-based vehicles are particularly attractive for delivery of bio-therapeutic agents that are difficult to synthesize, have reduced half-lives, limited tissue penetration or are rapidly inactivated upon direct *in vivo* introduction. As a matter of facts, the cell-based delivery system possesses a number of advantages including prolonged delivery times, targeting of drugs to specialized cell compartments and biocompatibility. The use of a physiological carrier to deliver therapeutics throughout the body to both improve their efficacy while minimising inevitable adverse side effects, is an appealing perspective that can be applied in many clinical settings. The behaviour of blood cells, as a delivery system for several classes of molecules (i.e., proteins, including enzymes and peptides, therapeutic agents in the form of nucleotide analogues, glucocorticoid analogues), has been studied extensively as they possess several properties, which make them unique and useful carriers [18]–[20]. In the context of extracorporeal pharmacotherapy, the most promising approach is the use of autoerythrocytes which possess unique morphological and physiological characteristics. Application of erythrocytes as containers for various drugs decreases the risk of side effects and pathologic immune reactions against encapsulated agents and, moreover, it allows the modification of their pharmacokinetic and pharmacodynamic properties for reducing doses and intervals between them. The efficacy and safety of pharmaceutical agents introduced into erythrocytes have been confirmed in numerous *in vivo* studies [20]. The magnetic erythrocytes, resulting from the co-encapsulation of the drugs with some ferrofluids such as cobalt–ferrite and magnetite, have been reported to direct the encapsulated drug predominantly to the desired sites of the body by means of an external magnetic field [21]–[23]. Applying ultrasound waves in the region where magnetic erythrocytes accumulate can induce vehicle destruction and the release of a drug at the organ or tissue level [24], [25]. In addition to drug targeting, this method has been evaluated for induction of local ischemia in tumors, which may consequently help to promote tumor remission because of reduced local blood flow [22]. However, the potential ability of cell carriers to provide site-specific or targeted delivery of therapeutics has yet to be fully explored. Development of cell targeting strategies will further advance cell vehicle applications, broaden the applicability of this delivery approach and potentiate therapeutic outcomes.

In an attempt to improve the targeting and the efficient release of therapeutic compounds at the intra-cellular level of target cells, we have developed a novel magnetic field-controlled erythrocyte-based drug delivery system. Attaching a viral spike fusion glycoprotein on the erythrocytes' membrane and encapsulating superparamagnetic nanoparticles and drug into erythrocytes, we have produced new erythrocytes-based drug delivery system, the erythro-magneto-HA virosomes, which have the potential for magnetic-controlled site-specific localization and highly efficient fusion capability with the targeted cells. The presence of viral fusion glycoprotein makes the application of ultrasound wave unnecessary for inducing “vehicle” destruction for the release of therapeutic compounds at the target site. Here we show how the erythro-magneto-HA virosomes behave somewhat akin to an enveloped virus in their ability to invade host cells. These engineered erythrocytes are able to fuse with the cytoplasmatic membrane of target cells in a very short time and release the magnetic nanoparticles and therapeutic compound inside these cells. The total amount of the anticancer drug 5-Aza-2-deoxycytidine has a therapeutic effect 10 times less than that of standard therapy, suggesting that this new drug delivery system may be able to reduce cytotoxic effects of drugs by increasing the bioavailability of therapeutic compounds at the site of action and improving pharmacokinetics. This new erythrocyte-based drug delivery system can potentially be applied in many clinical settings, including neoplastic pathologies, pathologies caused by the infection of a human or animal viruses and cardiovascular diseases.

## Materials and Methods

### Influenza Virus growth and isolation of Hemagglutinin (HA) glycoprotein

Influenza virus obtained from the allantoic liquid of 10-days-old embryonated eggs (eggs were obtained from chicken factory in Rome, Italy) was pelleted by ultracentrifugation and the pellet was resuspended in octa(ethyleneglycol)-*n*-dodecyl monoether (C_12_E_8_) and left overnight to allow for complete solubilisation of the viral membrane. The suspension was carefully homogenised and ultracentrifugated to pellet down the viral nucleocapsids. Next, the virosome suspension (supernatant containing the HA protein) was purified by ultracentrifugation on a discontinuous sucrose-gradient (50% and 10% of sucrose). At the interface of the sucrose layers, the HA glycoprotein concentrates. After removal of this layer, the HA has been dialysed against buffer and sterilized by filtration.

### Erythrocytes lysis and incorporation of HA glycoprotein, super paramagnetic nanoparticles and 5-Aza-2-dC drug

Human Red Blood Cells (RBCs) were obtained from blood of healthy donors by centrifugation at 1700 rpm for 10 min at 4°C. Freshly collected RBCs were washed twice in physiological phosphate-buffer saline (1X PBS) (1.37 M NaCl, 57 mM KCl, 54 mM Na_2_HPO_4_, 45 mM KH_2_PO_4_ pH 7.4).

2×10^9^ RBCs were lysed in 300 µl lysis buffer (10 mM TRIS, 0.1 mM EDTA, 1 mM MgCl_2_ at pH 7.2) for 60 minutes at 0°C. The isotonicity was then restored by adding 65 µl of 1 X resealing buffer (1.37 M NaCl, 57 mM KCl, 54 mM Na_2_HPO_4_, 45 mM KH_2_PO_4_, 15 mM MgCl_2_ pH 7.4), supplemented with 0.1 mg of 100 nm red-labelled superparamagnetic nanoparticles (nano-screenMAG, Chemicell Company), 1 µg HA influenza viral spike glycoprotein, and 0.38 or 30.5 µg 5-Aza-2-dC (Sigma-Aldrich, St Louis, MO, USA).

The suspension was incubated for 45 minutes at 37°C under mild agitation. Sealed RBCs (ghosts), containing HA fusion protein on lipid membrane and both 5-Aza-2-dC and superparamagnetic nanoparticles inside (erythro-magneto-HA virosome), were collected by centrifugation at 10,000 rpm for 15 minutes at 4°C. The erythro-magneto-HA virosomes were washed twice with 1X PBS by centrifugation at 9,000 rpm for 15 minutes at 4°C and resuspended in 1X PBS containing 1% FCS.

### Kinetics of Erythro-magneto-HA virosomes fusion with HeLa cells: Spectrofluorimeter analysis

Erythro-magneto-HA virosomes were prepared as described above and were labelled by adding Octadecyl Rhodamine (R18). The Erythro-magneto-HA virosomes/R18 loaded were added to HeLa cells. Total lipid in erythro-magneto-HA virosomes/R18 was quantified by the amount of Octadecyl Rhodamine (R18) in their membrane, based on the fact that R18 accounts for 15% of the total weight of lipids on it. The erythro-magneto-HA-virosomes/R18 were solubilized by adding 0.1% (final concentration) Triton X-100 in PBS, and the fluorescence of R18 (excitation at 560 nm and emission at 590 nm) was measured using an aliquot of the solution with a spectrofluorometer Perkin Elmer 650-40), calibrated with R18 standard solutions containing 0.1% Triton X-100. The degree of R18 self-quenching in each erythro-magneto-HA virosomes was examined by comparison of R18 fluorescence before and after solubilization of the erythro-magneto-HA virosomes with 0.1% Triton X-100 in PBS. Erythro-magneto-HA virosomes were added to HeLa cells. The kinetic of erythro-magneto-HA virosomes fusion with HeLa cells was calculated as % of R18 fluorescence dequenching (FDQ) using 560 and 590 nm as excitation and emission wavelengths respectively. As control we prepared R18-labeled magnetic erythrocytes without reconstituted HA fusion protein. The kinetic of fusion of these magnetic erythrocytes with HeLa cells was also analyzed.

### HPLC analysis of 5-Aza-2-dC incorporation in Erythro-magneto-HA virosomes

#### Chemicals and solutions

5-Aza-2-deoxycytidine (5-Aza-2-dC, Decitabine) was purchased from Sigma-Aldrich (St Louis, MO, USA). Ammonium acetate was from Carlo Erba (Milan, Italy). HPLC grade acetonitrile (MeCN) was from Rathburn (Walkerburn, UK).

#### HPLC analysis of Erythro-magneto-HA virosomes containing decitabine

2×10^9^ erythro-magneto-HA virosomes were incubated with 200 µl lysis buffer to release the incorporated 5-Aza-2-dC drug and centrifuged at 10,000 rpm for 15 minutes at 4°C. The supernatant was recovered and used for HPLC analysis or stored in a −80°C freezer until analysis.

Stock standard solutions of decitabine at concentration of 114, 228, 456 and 1140 ng/ml, were prepared individually in water solution or lysis buffer and stored at −80°C until analysis.

The peak area ratio of sample/5-Aza-2-dC (decitabine, standard) was used for quantitation. The limit of detection (LOD) was defined as three times the signal-to-noise ratio. The lowest limit of quantitation (LLOQ) was defined as the lowest level of analyte that could be reliably detected and reproducible with a precision of ≤20% and accuracy of 80–120%.

#### Instrumentation

The HPLC system comprised of a Dionex (Sunnyvale, CA, USA) 3000 Ultimate series LC connected to a linear ion Trap LTQ-Orbitrap (Thermo Fisher Scientific, USA) mass spectrometer, equipped with an electrospray ion source. Data were acquired and processed with Excalibur 2.1 software. Compounds were separated on a Gemini C18 (150 mm-2.0 mm I.D.) and 3 µm particle size (Phenomenex, Torrance, CA, USA) protected by a Phenomenex Gemini C18 (4.0 mm—2.0 mm I.D.) and 3 µm particle size guard cartridge. The HPLC method used gradient elution; mobile phase solvent A was water with 0,1% formic acid and mobile phase B was acetonitrile with 0,1% formic acid. The initial mobile phase composition of 92% solvent A and 8% solvent B was maintained for 2 min. Between 2 and 9 min the percentage of mobile phase B was increased to 35% and then back to initial the mobile phase composition within 0,1 min, with a total time of 14 min. The column was set at a flow rate of 0.2 ml min−1 and a temperature of 36°C. Sample volume of 10 µl was used for all LC–MS experiments. The mass spectrometer was operated in positive electrospray mode. The capillar temperature was 275°C and the spray voltage 4.5 kV was used.

### Tumor HeLa cells treatments

Human HeLa cells were purchased from the American Type Culture Collection (Rockville, MD).

The cells were maintained in DMEM medium supplemented with 10% fetal bovine serum, 2 mM L-glutamine, at split ratio of 1∶4 twice a week.

For treatments, 3 ml of cells at a density of 5×10^5^ were seeded in 6-well microtiter plates. Two sets of experiments were run in parallel for both Confocal Laser Scanning Microscopy (CLSM) and FACS analysis. For CLSM analysis, on the bottom of the culture plates there were placed glass coverslips on which the cells were let grow.

After 24 hours, the culture medium was changed with media containing 2,5 µM 5-Aza-2-dC alone or 1×10^9^ 5-Aza-dC-loaded erythro-magneto-HA virosomes or buffer in which the erythro-magneto-HA-virosomes were resuspended (supernatant, control-2). After 30 min, 1, 6, 24, 48 and 96 hours of incubation, treated and untreated (control-1) cells were analyzed by CLSM and FACS analysis.

### Confocal Laser Scanning Microscopy (CLSM) analysis of fusion events of erythro-magneto-HA virosomes with tumor cells

Tumor cells grown on slides in presence of 5-Aza-2-dC-loaded erythro-magneto-HA virosomes were washed in 1X PBS buffer and fixed with 4% paraformaldehyde. Cell nuclei were counterstained with DAPI and mounted in antifade medium. Fluorescence and bright-field images were obtained by a Leica TCS SP5 inverted microscope system, equipped with five lasers emitting from the UV to the visible (405 Diode, Argon, HeNe 543, HeNe 594 and HeNe 633). The DAPI fluorescence was detected at excitation of 405 nm and emission of 454 nm while the red fluorescence of superparamagnetic nanoparticles at excitation of 543 nm and emission of 613 nm.

### Cytofluorimetric analysis (FACS analysis)

FACS analysis was carried out on HeLa cells treated with 2,5 µM 5-Aza-dC or 1X 10^9^ 5-Aza-2-dC-loaded erythro-magneto-HA-virosomes and compared to those untreated (control-1) after 24, 48 and 96 hours of culture. FACS analysis was also carried out on HeLa cells treated with buffer in which the erythro-HA-virosomes were resuspended to check the absence of unincorporated 5-Aza-2-dC drug (control-2). Treated and untreated cells were fixed in ethyl alcohol and the nuclei were stained with 10 µg/ml of propidium iodide and incubated with 100 µg/ml of RNase for 1 hour at 37°C. The nuclear DNA content, which discriminates the cell cycle phases, was determined by flow cytometry using the Becton–Dickinson FACScan CentroII.

### Ethics statements

For the present study we did not need ethic approval since the work was not involving human nor animal studies.

The present study was performed without utilizing donors from human volunteer material, human blood was obtained from transfusional bag from the blood bank, for those reasons we did not need informed consent.

## Results

### Behaviour of erythro-magneto-HA virosomes

**Figure 1:**
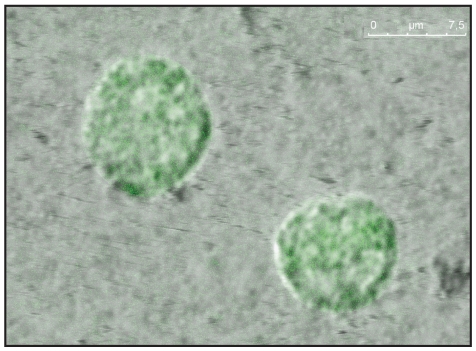
Engineered Erythrocytes. Confocal Laser Scanning Microscopy (CLSM) images of erytro-magneto-HA virosomes encapsulating 100 nm fluorescence-labelled superparamagnetic nanoparticles (green) and 5-Aza-2-dC.

A new erythrocyte-based drug delivery system described here is potentially capable of delivering drugs to target tissues increasing the bioavailability of therapeutic compounds at the site of action leading to a better therapeutic effect with minimal side effects. Human erythrocytes were isolated from the blood of a healthy donor and treated with a hypotonic solution to induce the opening of membrane pores and release hemoglobin. A mix containing Hemagglutinin (HA) viral spike fusion proteins, fluorescent superparamagnetic nanoparticles and the therapeutic compound was added during the resealing of “ghost” erythrocytes. Due to the high affinity for membranes, hemagglutinin binds to the cytoplasmatic membrane of erythrocytes while magnetic nanoparticles (green fluorescence) and drug penetrate through pores into “ghost” erythrocytes (**Fig. 1**).

**Figure 2:**
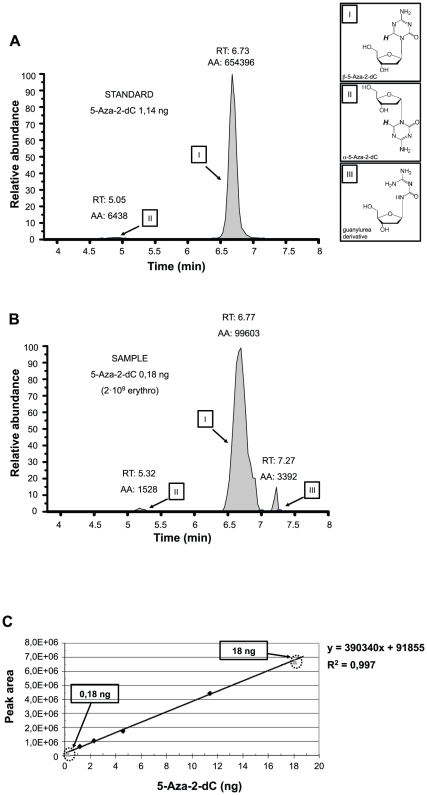
HPLC/QTOF quantitative analysis of 5-Aza-dC. (**A**) Peaks of 1,14 ng 5-Aza-dC (standard). (**B**) Peaks of 5-Aza-dC encapsulated into 2×10^9^ erythro-magneto-HA virosomes. **RT**: retention time; **AA**: peak area counts. (**C**) Calibration curve of 5-Aza-dC. The peak area values of standards were put in relation with the ng/10 µl of 5-Aza-2-dC sample ran. Standard solutions used were 1,14, 2,28, 4,56 and 11,40 ng 5-Aza-dC (black rhombuses). Grey triangles show the 5-Aza-dC concentrations found within the two erythro-magneto-HA virosomes samples used in *in vitro* experiments.

To verify the effective trapping of 5-Aza-2-dC (Decitabine) in our erythrocyte-based drug delivery system and to quantify the yield of therapeutic compound encapsulated, we performed HPLC analysis (**Fig. 2**). Liquid chromatography/tandem mass spectrometry quantification method (QTOF/MS) has been used in order to quantify and identify the 5-Aza-2-dC isoforms and their decomposition products under physiologic conditions of pH and temperature as previously described [26], [27].

In **Figure 2** **A** and **B**, the peaks of both 5-Aza-2-dC (standard) and 5-Aza-2-dC loaded in 2×10^9^ erythro-magneto-HA virosomes are shown. The samples were monitored in positive mode, resulting in species of 1 mass unit higher than the neutral molecules. Data were visualized by extracting the ion chromatograms at m/z = 229. In the standard solution (**Fig. 2** **A**) two peaks corresponding to both single charged β- (I) and α-forms (II) of 5-Aza-2-dC (m/z = 229) are present. In the sample, another peak is evident in addition to 5-Aza-2-dC β- and α-forms (I and II). This peak corresponds to a guanylurea derivate (III) (m/z = 219). As previously shown, extracted ion chromatograms at m/z = 219 (guanylurea derivatives, single charged monomeric species) are not unique as an identifier of guanylurea derivatives as compounds with m/z = 229 also produce fragments with m/z = 219 when visualized by mass spectrometer [28]. β-forms (I) of 5-Aza-2-dC become hydrated at the C-6 position and the ring-opens to produce a ring-open-formylated derivative, followed by loss of formic acid, which then produces a guanylurea derivative (III). Deformylation of any of the ring-open formylated derivatives into the guanylurea derivatives (III) results in loss of the H-6 proton as formic acid. The retention times (RT) as well as the area values (AA) for each peak are shown in **Figure 2** **A** and **B**.

To quantify the amount of 5-Aza-2-dC contained in the erythrocyte-based delivery system, 10 µl of each standard and sample were injected in HPLC. Different concentrations of standard, spanning from 0,114 ng/µl (0,5 µM) to 1,14 ngµl (5 µM) of 5-Aza-2-dC, were prepared and the peak area of each 10 µl sample was calculated by HPLC. The peak area values of standards were put in relation to the ng of 5-Aza-2-dC and a calibration curve was designed (**Fig. 2** **C**). **Figure 2** **A** shows the area value (AA: 660.834) detected by HPLC corresponding to 1,14 ng (0,5 µM) of 5-Aza-2-dC standard solution. Different samples of erythro-magneto-HA virosomes were prepared and different amounts of 5-Aza-2-dC, spanning from 0,5 ng/µl (2,5 µM) to 42 ng/µl (200 µM), were added during the resealing. To assess the effective amount of 5-Aza-2-dC entrapped in erythrocytes for each preparation, 2×10^9^ erythro-magneto-HA virosomes were lysed in 200 µl lysis buffer. The total amount of drug incorporated into 2×10^9^ erythro-magneto-HA virosomes was 3,6 ng or 360 ng when 2,5 µM or 200 µM of 5-Aza-2-dC were added to erythrocytes during resealing, respectively (**Fig. 2** **C**). Therefore, the efficiency of incorporation for each sample was approximately 1%.

**Figure 3:**
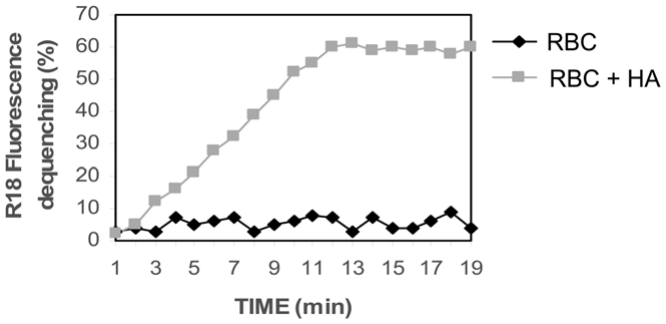
Kinetic Fusion assay. The 5-Aza-2-dC-loaded erythro-magneto-HA virosomes were labelled with Octadecyl Rhodamine (R18) and incubated with HeLa cells. Fusion has been reported as % of R18 fluorescence dequenching (FDQ) using 560 and 590 nm as excitation and emission wavelengths respectively. RBC: 5-Aza-2-dC-loaded magnetic erythrocytes without reconstituted HA fusion protein (Control); RBC-HA: 5-Aza-2-dC-loaded erythro-magneto-HA virosomes.

To test the effective incorporation of Hemagglutinin (HA) on erythrocyte membranes and its fusion activity, we evaluated the kinetics of fusion of our engineered erythro-magneto-HA virosomes with target cells. Octadecyl Rhodamine (R18) labeling was used to determine the fusion of erythro-magneto-HA virosomes with HeLa cells. Fusion has been reported as % of R18 fluorescence dequenching (FDQ) using 560 and 590 nm as excitation and emission wavelengths, respectively. The fusion of erythro-magneto-HA virosomes with HeLa cells starts after a very short period (few minutes) and the percentage of fusion exponentially increases with time (**Fig. 3**). In contrast, magnetic erythrocytes without Hemaglutinin (HA) do not show fusion activity with HeLa cells. These data indicate that the viral spike glycoprotein (HA) purified from influenza virus and reconstituted in magnetic erythrocyte vehicles maintains its fusogenic properties with the cytoplasmatic membranes of the target cells and confers on magnetic erythrocytes the ability to fuse with target cells.

### Effect of Decitabine-loaded erythro-magneto-HA virosomes on tumor cells

To verify the efficiency and efficacy of our erythrocyte-based drug delivery system, we treated HeLa tumor cells either with 5-Aza-2-dC alone or encapsulated into erythro-magneto-HA virosomes. 5×10^5^ tumor cells were treated either with 1.800 ng of 5-Aza-2-dC (2,5 µM), a concentration shown to have therapeutic effect *in vitro*, or with 1×10^9^ erythro-magneto-HA virosomes containing 10 times less 5-Aza-2dC (180 ng).

**Figure 4:**
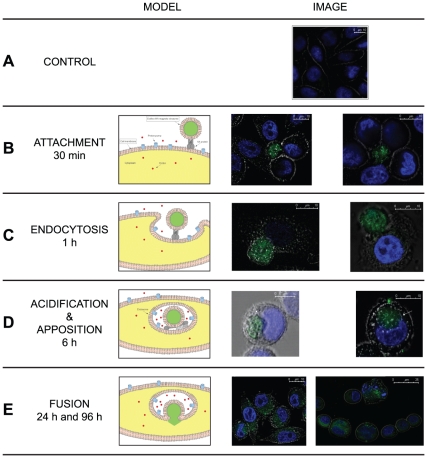
Confocal Laser Scanning Microscopy (CLSM). In the left is schematized the timing and mechanism of action of the erythro-magneto-HA virosomes, encapsulating 100 nm fluorescence-labelled magnetic nanoparticles (green) and 5-Aza-2-dC. In the right are shown CLSM images of erythro-magneto-HA virosomes (green) after 30 minutes (**A**), 1 hour (**B**), 6 hours (**C**), 24 and 96 hours (**D**) of incubation with HeLa cells highlighted by DAPI staining (blue). (**CTRL**) Untreated HeLa cells (control).

The uptake to the target cell and delivery of the therapeutic compound into the cytoplasm of tumor cells is shown in **Fig. 4**. At very short times (from 30 min to 1 hour) it is still possible to distinguish engineered erythrocytes which start to blend with target cells (**Fig. 4** **A** and **B**). After 6 hours most of the target cells show the erythro-magneto-HA virosomes within the cytoplasm (**Fig. 4** **C**). The erythro-magneto-HA virosome, internalized inside the “host” cell, looks like a viral endosome. The membrane of erythro-magneto-HA virosomes starts to fuse with the membrane of the target cell between 12 and 24 hours, whereupon the fluorescent nanoparticles and the drug start to diffuse inside the tumor cells. After 24 hours all the tumor cells showed a strong fluorescence in the cytoplasm and some have the typical morphology of early apoptosis. At 96 hours most of tumor cells show the characteristic micronuclei morphology of late apoptosis (**Fig. 4** **D**).

**Figure 5:**
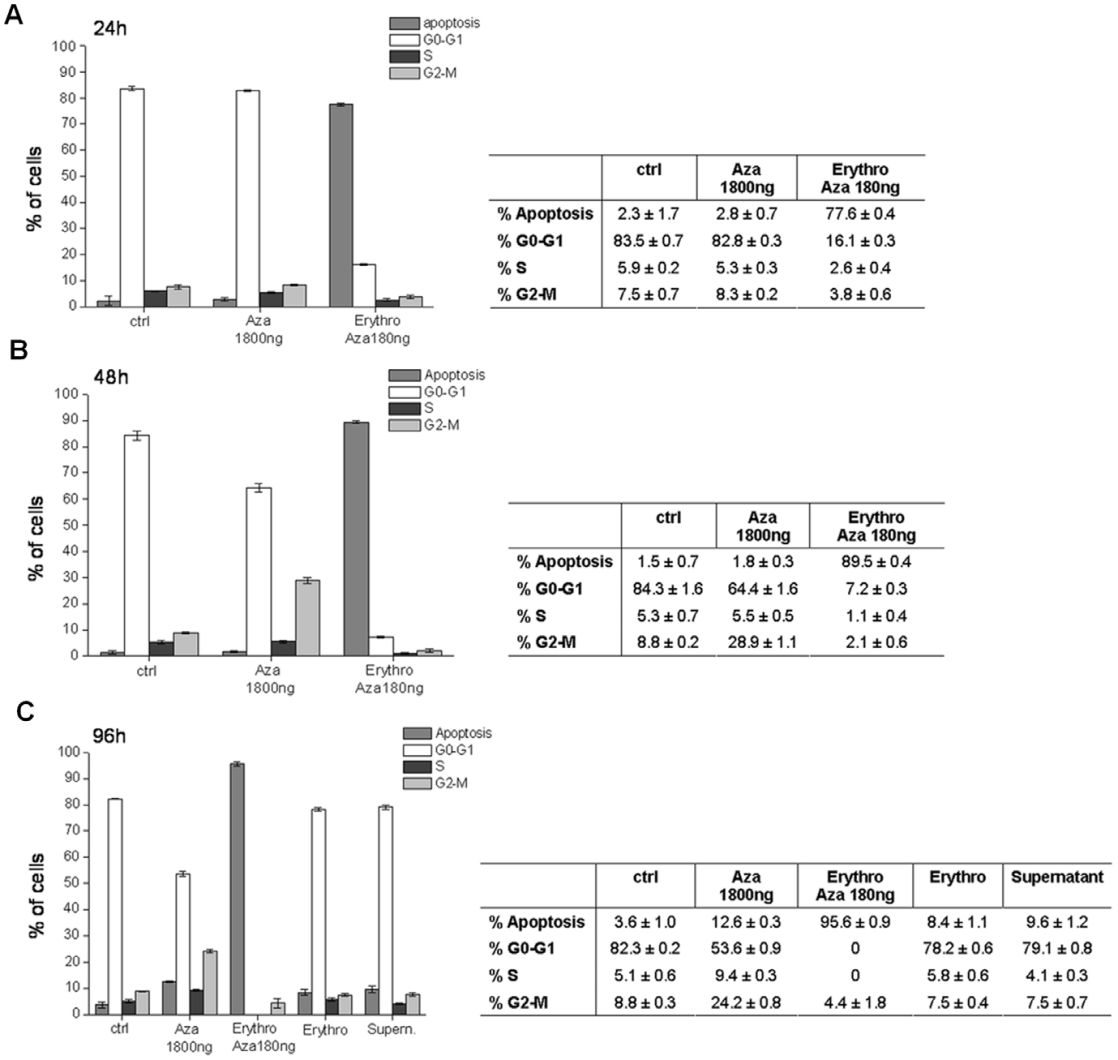
FACS analysis of HeLa cells after (A) 24 h, (B) 48 h and (C) 96 h treatments. **Ctrl** (control) untreated cells; **Aza:** cells treated with 1800 ng (2,5 µM) of 5-Aza-2-dC; **Erythro Aza:** cells treated with erythro-magneto-HA virosomes containing 180 ng of 5-Aza-2-dC; **Erythro:** unloaded 5-Aza-2-dC erythro-magneto-HA virosomes; **Supernatant:** cells treated with buffer where the erythro-magneto-HA virosomes were resuspended (control 2).

The efficacy of 5-Aza-2-dC reconstituted into erythro-magneto-HA virosomes in promoting apoptosis relative to free drug is shown in **Figure 5**. HeLa cells untreated (control) and treated with 1800 ng 5-Aza-2-dC or with 1×10^9^ erythro-magneto-HA virosomes containing 180 ng of Decitabine were analyzed by FACS. As controls to assess possible toxic effects of the vehicle alone on cells, HeLa cells were cultured also in presence of the buffer solution in which 5-Aza-2-dC-loaded erythro-magneto-HA virosomes were suspended (supernatant) and with erythro-magneto-HA virosomes encapsulating only the fluorescent magneto-nanoparticles.

After 96 hours only 12,6% of cells treated with 1800 ng of free 5-Aza-2-dC appear apoptotic compared with 96% of cells when cells are treated with 180 ng in erythro-magneto-HA virosomes. Moreover, at 24 hours significant apoptosis is seen with the drug delivery system while virtually no effect is observed for drug alone. These observations suggest that erythro-magneto-HA virosomes delivery system increases the anti-neoplastic effect of 5-Aza-2-dC through enhancement of drug bioavailability. Neither the supernatant, where the 5-Aza-2-dC-loaded erythro-magneto-HA virosomes are suspended, nor erythro-magneto-HA virosomes containing only fluorescent nanoparticles have an effect on cell proliferation, demonstrating that our erythrocyte-based drug delivery system itself has not toxic effect on cells. Together these results suggest that this new erythrocyte-based drug delivery system has the potential to increase the efficiency and efficacy of therapeutic compounds.

## Discussion

Cytotoxic chemotherapy or radiotherapy of cancer is limited by serious, sometimes life-threatening, side effects because of the lack of specificity to target cells alone. One strategy to improve specificity is to encapsulate the therapeutics into a drug delivery system capable of directing therapeutic compounds to a target site.

Carrier erythrocytes have been evaluated for their use in drug delivery in humans proving their safety and efficacy [29]. Erythrocyte-based delivery of new and conventional drugs is thus experiencing increasing interests in drug delivery and in managing complex pathologies, especially when side effects could become serious issues [13], [19], [30]. Recently, erythrocytes have been used to encapsulate the antibiotic amikacin, and a sustained release from loaded erythrocytes over a 48 hours period was demonstrated, suggesting a potential use of the erythrocytes as a slow systemic-release system for antibiotics. The administration of amikacin by loaded erythrocytes in rats leads to significant changes in the pharmacokinetic behavior of the antibiotic [31], [32]. The erythrocyte-based drug delivery system presents various advantages: it is especially efficient in releasing drugs in the circulation for long period of time (weeks), erythrocytes have a large trapping capacity, are easily processed and have capability to accommodate traditional and biologic drugs [18], [30]. Erythrocytes loaded with ferromagnetic colloid compound can be magnetically driven at the target organ/tissue by means of an external magnetic field [21]–[23], [33] Finally, the availability of an apparatus that permits the encapsulation of drugs into autologous erythrocytes makes this technology available in many clinical settings and competitive with respect to other drug delivery systems [34].

In the present work we point out a new erythrocyte-based drug delivery system, the erythro-magneto-HA virosome, where the human erythrocytes have been engineered with both viral spike fusion proteins and superparamagnetic nanoparticles. This drug delivery system, thanks to its both magnetic and high efficient fusion properties, can be used to specifically drive the drug or pro-drug at target organ or tissue and release the therapeutic compound directly inside the target cells thus improving the efficiency and efficacy of therapy.

The efficiency of our erythrocyte-based delivery system based on the possibility to magnetically drive and concentrate the therapeutic compounds in a site-specific manner by applying physical method, the static magnetic field, at level of target tissue/organ, as previously described [21]–[23], [33]. In this way it is possible to control the distribution and location of the drug delivery system because of the magnetic properties of nanoparticles encapsulated inside the red blood cells. Furthermore, the efficiency of our system also depends on its high capability to fuse with the target cells through the binding of hemagglutinin (HA) viral spike fusion protein to the target cell membrane thus improving the efficacy of therapy. Kinetic fusion assay data outlined here indicate that the hemagglutinin (HA) viral spike glycoprotein, purified from influenza virions and reconstituted in magnetic erythrocyte vehicles, maintains its fusogenic properties thus determining the integration of the delivery system with the cytoplasmatic membranes of target cells notwithstanding the dimension (8 micron) of the erythro-magneto-HA virosomes.

Our observations indicate that the engineered erythrocytes dock onto the target cell through the interaction of the HA protein with sialic acid residues on the surface of the target cells. The integration of the erythrocytes into target cells presumably occurs through receptor-mediated endocytosis. The erythrocytes presumably enter endosomes where subsequent fusion of erythrocyte and endosomal membranes releases drug and magnetic particles into the cytoplasm. In this manner, the drug encapsulated in the “ghost” erythrocytes does not undertake its pharmacological and toxicological function until it reaches the target cells. This is of considerable importance in the case of certain drugs with short half-life or with major toxicity such as anti-neoplastic drugs.

Here we show that the pharmacological and toxicological function as well as half-life of one test drug, 5-Aza-2-dC, are preserved and enhanced by our erythrocyte-based delivery system.

5-Aza-2-dC is effective against myelodysplastic syndromes and various types of leukaemias [35]–[37] and is in clinical trial for other human cancers [38], [39]. The primary mechanism for the activity of 5-Aza-2-dC is believed to be through inhibiting methyltransferases involved in epigenetic modifications [40] 5-Aza-2-dC also has demonstrated toxicity [41] and some reports suggest that it could be mutagenic [42]. The 5-Aza-2-dC is a pro-drug that requires metabolic activation by deoxycytidine kinase to exert its function as anti-cancer drug [35]. In human, 5-Aza-2-dC has a short half-life of 15 to 25 minutes due to rapid inactivation by liver cytidine deaminase. The instability of 5-Aza-2-deoxycytidine under physiological conditions creates a significant challenge to its use in humans. It has previously been reported that a plethora of compounds are generated during the chemical decomposition of 5-Aza-2-dC at physiological pH and temperature [28], [43]. It has been shown that 5-Aza-2-dC is converted into its corresponding ring-open-formylated derivates followed by irreversible deformylation into the guanylurea derivates. It has been suggested that these ring-open products block DNA polymerases and create potentially lethal lesions [44]. The guanylurea derivates have the potential to mispair during replication thus determining an accumulation of mutations into DNA. The chromatography/tandem mass spectrometry quantification method (see the QTOF/MS analysis) is used here to quantify and identify the 5-Aza-2-deoxycytidine isoforms and its eventual decomposition products. Our results indicate that the erythro-magneto-HA virosomes may protect the 5-Aza-2-dC from rapid and premature decomposition thus leading to an improvement of half-life of therapeutic compound. Moreover, data from FACS analysis indicate that the total amount of this anti-cancer drug necessary to obtain growth arrest and apoptotic response in tumor cells is approximately 10 times less when erythro-magneto-HA virosome delivery system is used, compared to standard therapy suggesting that the erythro-magneto-HA virosomes highly increase the bioavailability of the drug. Furthermore, toxicity is observed at earlier times when the drug was encapsulated into erythro-magneto-HA virosomes.

### Conclusion

In conclusion we report here a novel erythrocyte-based drug delivery system, the erythro-magneto-HA virosomes, which present many potential advantages that enable it to be used in certain situations as an alternative to other drug delivery systems. This advantages include: enhancing the bioavailability of drugs through its high fusogenic capability with target cells; the potential for magnetically driving the virosomes to desired sites of action; its potential for protecting therapeutics from degradation or elimination prior to reaching target tissues or organs; its high biocompatibility relative to other carrier systems which may be optimized by employing autologous erythrocytes. Finally, the composition of this erythrocyte-based drug delivery system make it applicable to many clinical settings, such as neoplastic and cardiovascular diseases, pathologies caused by the infection of a human or animal virus and certain metabolic diseases.
